# Real-Time Control of a Multi-Degree-of-Freedom Mirror Myoelectric Interface During Functional Task Training

**DOI:** 10.3389/fnins.2022.764936

**Published:** 2022-03-11

**Authors:** Andrea Sarasola-Sanz, Eduardo López-Larraz, Nerea Irastorza-Landa, Giulia Rossi, Thiago Figueiredo, Joseph McIntyre, Ander Ramos-Murguialday

**Affiliations:** ^1^Neurotechnology Unit, TECNALIA, Basque Research and Technology Alliance, Donostia-San Sebastian, Spain; ^2^Institute of Medical Psychology and Behavioral Neurobiology, University of Tübingen, Tübingen, Germany; ^3^Bitbrain Technologies, Zaragoza, Spain

**Keywords:** motor learning, myoelectric interface, multi-DoF exoskeleton control, rehabilitation, stroke

## Abstract

Motor learning mediated by motor training has in the past been explored for rehabilitation. Myoelectric interfaces together with exoskeletons allow patients to receive real-time feedback about their muscle activity. However, the number of degrees of freedom that can be simultaneously controlled is limited, which hinders the training of functional tasks and the effectiveness of the rehabilitation therapy. The objective of this study was to develop a myoelectric interface that would allow multi-degree-of-freedom control of an exoskeleton involving arm, wrist and hand joints, with an eye toward rehabilitation. We tested the effectiveness of a myoelectric decoder trained with data from one upper limb and mirrored to control a multi-degree-of-freedom exoskeleton with the opposite upper limb (i.e., mirror myoelectric interface) in 10 healthy participants. We demonstrated successful simultaneous control of multiple upper-limb joints by all participants. We showed evidence that subjects learned the mirror myoelectric model within the span of a five-session experiment, as reflected by a significant decrease in the time to execute trials and in the number of failed trials. These results are the necessary precursor to evaluating if a decoder trained with EMG from the healthy limb could foster learning of natural EMG patterns and lead to motor rehabilitation in stroke patients.

## Introduction

A voluntary movement is the result of complex mechanisms in which the central nervous system recruits groups of muscles in a coordinated way, with different activation patterns and temporal profiles that are encoded at the spinal or brainstem levels (Tresch et al., 1999; d’Avella et al., 2003; Bizzi et al., 2008; Bizzi and Cheung, 2013). Local brain damage due to stroke frequently affects the initiation of motor commands and/or their descending flow to the spinal cord. This leads to disrupted recruitment or to abnormal development of muscle-activation patterns and so, to pathological muscle coordination in the limbs opposite to the injured hemisphere. Motor recovery after stroke is characterized by neuroplastic changes involving a structural and functional reorganization of the brain (Shmuelof and Krakauer, 2011; Ramos-Murguialday et al., 2013). This implies the recruitment of intact cortical motor structures adjacent to the injury, which generate commands to the compromised muscles that are relevant for the intended task (Krakauer, 2006). However, the brain networks involved in motor learning and recovery processes and the underlying neural mechanisms are not yet well understood (Hosp and Luft, 2011; Takeuchi and Izumi, 2013). Nonetheless, there is evidence that the brain and the lower sensorimotor circuitry can change or reorganize itself in response to sensory input, experience and learning (Chan et al., 2006; Cramer et al., 2007; Kleim and Jones, 2008). A remaining challenge, however, is how to foster the relearning of natural muscle activation patterns in order to achieve effective motor recovery.

Motor leaning is a complex process that comprises motor adaptation and skill acquisition (Shadmehr and Wise, 2005; Krakauer, 2006; Krakauer and Mazzoni, 2011; Wolpert et al., 2011). Motor adaptation occurs implicitly, presumably over a short period and as a response to the error between what the brain predicts and the observed outcome. Motor skill acquisition is instead related to the ability to accurately execute the movement and may need a more extensive training period to occur (Kitago and Krakauer, 2013). The relationship of motor skill acquisition and adaptation to motor recovery is still unclear. Several studies have confirmed that functional recovery mediated by motor training entails a learning process in patients with motor impairment (Chan et al., 2006; Krakauer, 2006, 2015; Piron et al., 2010; Arya et al., 2011; Dipietro et al., 2012; Reinkensmeyer et al., 2016). However, training paradigms still need to be optimized to become truly effective (Krakauer, 2006; Arya et al., 2011). In recent years, technological advances and rehabilitation strategies have explored methods to elicit learning as a means to achieve motor recovery. For instance, rehabilitation robots allow intense task training, precise control of timing and the use of visual and proprioceptive feedback, which enhances motor learning (Kwakkel et al., 1997; Reinkensmeyer et al., 2004; Prange et al., 2006; Marchal-Crespo and Reinkensmeyer, 2009).

Electromyographic (EMG) signals have been widely explored for the control of rehabilitation robots, as they offer a direct measurement of the motion intention of a person (Scheme and Englehart, 2011; Song et al., 2013; Ison et al., 2016; Vujaklija et al., 2018). However, several factors have hindered the exploitation of myoelectric interfaces and held up its transfer to commercial applications (Ison et al., 2014a). One of the main problems is the lack of systems that can simultaneously control several degrees of freedom (DoFs) in real-time. In the last few years, some studies have expanded from simple cursor control (Itou et al., 2001; Radhakrishnan et al., 2008) to the simultaneous control of multi-DoF external (non-wearable) robots in real-time (de Rugy et al., 2013; Pistohl et al., 2013; Ison and Artemiadis, 2015; Ison et al., 2016). However, the control of wearable prostheses or exoskeletons adds one more level of complexity, since human motor control mechanisms are difficult to model and mechanical constraints and dynamics of the robot might hinder control proficiency (Sartori et al., 2018). For these reasons, myoelectric interfaces for controlling wearable robots have mostly been validated for two DoF control, with only one example of a higher-order system with five DoFs simultaneously to perform up to three pairs of motions (Fougner et al., 2014; Tang et al., 2014; Muller-Putz et al., 2015; Amsuess et al., 2016; Lu et al., 2017).

Another concern in myoelectric applications is that most systems are usually validated in one single session, or those with longer paradigms are focused on recalibrating or adapting the mapping every new session as a way to optimize the learning process. However, Ison and colleagues recently demonstrated that using a fixed mapping between the EMG and the output control command could induce learning and the creation of novel muscle activation patterns or synergies in healthy individuals. Moreover, these synergies were retained after 1 week, facilitating the generalization to new tasks and the increase in performance over time without the need of recalibrating the decoder (i.e., changing the mapping) (Antuvan et al., 2014; Ison et al., 2014b; Ison and Artemiadis, 2015). This implies that the utilization of dynamic mappings may not be as critical as suggested in recent research. Based on these studies, we believe that hemiplegic stroke patients could reshape the pathological muscle activity of their paretic limb by learning a fixed mapping or model built with EMG activity of their healthy upper limb (i.e., a mirror myoelectric decoder), as suggested in Sarasola-Sanz et al. (2018). Whether such changes in the muscle activation patterns of the impaired limb of stroke patients would lead to functional recovery has not been proven yet. However, we hypothesize that reducing agonist-antagonist muscle-pair co-activations and spasticity through training with the mirror myoelectric decoder might yield a positive clinical outcome.

In this study, we investigated the viability of using a novel EMG decoding strategy to control an upper limb multi-joint exoskeleton in real-time during functional tasks, based on a mirror model from the contralateral arm (Sarasola-Sanz et al., 2018). The potential of this paradigm for the continuous decoding of multi-DoF functional movements was already proved offline (Sarasola-Sanz et al., 2015, 2017, 2018). Moreover, we evaluated if the proposed system can be used to elicit motor learning in 10 healthy participants and to adapt their muscle activity according to the imposed mirror EMG-to-kinematics map. Additionally, with a view toward optimizing such a system in a patient-centered approach to stroke recovery, we queried participants in the experiments about their perceptions of different features of the system.

## Materials and Methods

### Subjects

Ten able-bodied volunteers (five females and five males, age: 20–33, all right-handed) without any known neuromuscular impairment participated in this study. All of them gave written consent to the procedures as approved by the Ethics Committee of the Faculty of Medicine of the University of Tübingen, Germany.

### Experimental Setup and Protocol

**FIGURE 1:**
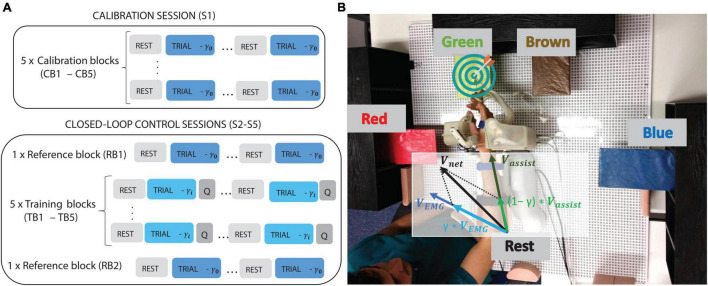
**(A)** Block structure of the calibration (S1) and closed-loop control sessions (S2–S5). The different assistance levels are indicated by different γ levels being γ_*0*_ = 0, trials with control independent of EMG (i.e., 100% assistance) and γ_*i*_ = 0.7, 0.4, or 0.9 (70, 40, or 90% EMG control, respectively). After each trial, participants were asked to rate how difficult they found it, (indicated by the Q letter), followed by rest intervals of 2–3 s. **(B)** Experimental setup indicating the four targets and the rest position, as well as the robotic exoskeleton and the way assistive (*V*_*assist*_) and EMG (*V*_*EMG*_) components of x- and y-DoFs were combined to compute the net component sent to the exoskeleton [see Equation (1)].

The IS-MORE robotic exoskeleton (Tecnalia, Donostia-San Sebastian, Spain) is a seven-DoF robotic exoskeleton for the proximal (upper and forearm) and the distal (wrist and fingers) segments of the upper limb [more details in Sarasola-Sanz et al. (2015)]. The exoskeleton allows movements in seven DoFs that are completely free and can take any position at any time: Movement and rotation of the forearm in a 2D horizontal plane (*p*_*x*_, *p*_*y*_, θ_*xy*_), pronation and supination of the wrist (ϕ_*wrist*_), and flexion and extension of the thumb (δ_*thumb*_), the index (ψ_*index*_) and the group of the middle, ring and pinky fingers (α_*3fingers*_), measured as the angle of rotation with respect to the metacarpophalangeal joints. The exoskeleton lies on a 70 cm × 50 cm mat on top of which the user performs gravity-compensated movements. Four shelves of different color placed around the mat define the four targets that the participants have to reach (see Figure 1B and Supplementary Video 1).

Volunteers were asked to sit on a chair in front of the workspace and wear the exoskeleton on their right or left upper limb for a single decoder calibration session and for four closed-loop control sessions, respectively. For each participant, a posture in which they could keep their arm relaxed was selected at the beginning of the calibration session and defined as the “Rest” position. Similarly, four target poses (i.e., the position and orientation of the arm around each target and the angle of the wrist and the fingers) were also defined according to each subject’s range of motion.

All the participants underwent five sessions on five consecutive days: the calibration session (S1) used to train an EMG decoder with the right arm, and four closed-loop control sessions (S2–S5) in which subjects performed targeted movements controlling the seven DoFs of the robot with EMG activity of the left arm, interpreted by the mirror decoder obtained in S1.

In the calibration session (S1) participants performed five blocks of “compliant movements” with the exoskeleton on their right arm, during which they had to follow the movement driven by the exoskeleton in an active way. They were constantly reminded to adhere to the pace and trajectory of the exoskeleton without counteracting the movement, although we could not control if this was really happening. The EMG was continuously visualized on a screen and checked by a therapist to control for co-contraction activity. However, we did not include any sensor that could quantitatively inform us about counteracting forces. We found the pace and movement range that felt comfortable for subjects and assumed that as the movement was repeated several times, they could get used to the pace and trajectories followed by the exoskeleton, which were not modified during the whole calibration session. These blocks of compliant movements with the right arm were referred to as calibration blocks (CBs; see Figure 1A). Each block was comprised of eight trials, each consisting of an outward (toward the target) and an inward (toward the “Rest” position) movement. Subjects were instructed to supinate the wrist and open their hand while approaching the target, and to pronate the wrist and close their hand while going back to the “Rest” position. Auditory cues were used to instruct subjects about the target position as well as to mark the beginning and end of each movement. An inter-trial rest interval of 3 s was included to avoid muscle fatigue and to allow the participants to prepare for the next trial. The data recorded during S1 was used to calibrate the myoelectric decoder.

In sessions S2–S5, participants performed two reference blocks (RBs), one at the beginning and one at the end of the session, and five training blocks (TBs) with their left arm. The RBs included compliant movements, the same as those performed during the calibration blocks, and served to help participants to get a reference for the trajectory the exoskeleton should ideally follow during the subsequent TBs. These trials also served to assess the adaptation of their EMG activity patterns as a result of the training within each session and along the intervention. In the TBs, the movement of the exoskeleton was determined by the weighted sum of two components [see Equation (1)]: a component based on the EMG activity of the subject, and an assistive component that would always redirect the exoskeleton toward the required position. From the eight trials included in each training block, six were randomly set to an assistance level of 30% (i.e., 70% of the velocity of the exoskeleton was based on EMG activity and 30% on assistive target directed velocities). The remaining two were catch trials assigned with assistance levels of 10 and 60% and randomly placed within the block of 8. The three different levels of assistance (catch trials plus main trials) served to evaluate the influence of the amount of assistance on the myoelectric control.

In every outward or inward movement, subjects were given 30 s to move as far as they could in the required direction. If they were unable to reach the target or the “Rest” position after the timeout, they would be presented with the next target. As a way of motivation, a piece of classical music with increasing intensity over time was played during each movement. Since the movements of the distal DoFs were finer and it was found that they were more difficult to control than the proximal DoFs (Sarasola-Sanz et al., 2015), the trials were considered to be completed when the target position of the three proximal DoFs (i.e., position and orientation of the arm *p*_*x*_, *p*_*y*_, and θ_*xy*_) was reached, independent of the angle of the wrist and the fingers. Nonetheless, even though the task accomplishment depended solely on the proximal DoFs’ position, participants were not informed of this fact. Subjects could control the movement in the seven-DoFs and received feedback in all of them. Therefore, in these training blocks, participants received visual and proprioceptive feedback of their EMG activity by means of the velocity modulation of the seven DoFs of the exoskeleton and an auditory reward if the target was reached.

After each trial, subjects were asked to rate the difficulty of the previous movement on a scale from 0 to 10, with 0 representing the lowest difficulty and 10 the highest. Similarly, the opinion of the participants regarding several aspects of the platform, such as the comfort of the setup, the difficulty of the task, the quality of the control and the functioning of the exoskeleton, was collected with a feedback questionnaire that they were asked to fill out at the end of each session.

### Data Acquisition and Processing

Two high-density arrays of 24 channels each (Tecnalia-Serbia, Belgrade, Serbia) were placed over the extensor and flexor muscles of the forearm to record the muscular activity with high spatial resolution. Differential signals were computed from each closest-neighbor pair of electrodes along the diagonals and axial directions, resulting in a total of 110 bipolar channels collected from the forearm muscles. In addition, six standard bipolar electrodes (Myotronics-Noromed, Kent, WA, United States) were used to record the EMG activity from the Abductor Pollicis Longus, the Biceps, the Triceps, and the Frontal, Middle and Posterior Portions of the Deltoid. The reference and ground electrodes were located over the olecranon and the clavicle, respectively. The location of the electrodes on the left and right upper limbs was symmetrical (e.g., the more medial electrodes on the right arm were matched with the corresponding more medial electrodes on the left arm). The positions on the left arm were marked with permanent markers to mitigate the effects of varying electrode positions across sessions.

The EMG activity was acquired at 1,000 Hz (Brain Products GmbH, Gilching, Germany), band-pass filtered (10–450 Hz), and comb-filtered (50 Hz and harmonics). Kinematics of the seven-DoFs were collected at 20 Hz and low-pass filtered (1.5 Hz).

### Feature Extraction and Decoder Calibration

Five time-domain features (mean of absolute value, variance, waveform length, root-mean-square error and the logarithm of the variance) were extracted from each EMG channel in windows of 200 ms. The resulting matrix of features was normalized to zero mean and unit variance using the mean and standard deviation computed from the whole calibration data set.

A channel selection process was applied to the high-density EMG channels to reduce the dimensionality of the feature input set. We followed an iterative cross-validation process (Sarasola-Sanz et al., 2017) to select a set of 10–50 channels. The kinematics collected during the calibration session were mirrored by flipping the sign of those DoFs that would have opposite directions for each arm (e.g., the sign of the velocity *v*_*x*_ along the *x*-axis toward the right or left side of the participant was flipped, while the velocity *v*_*y*_ along the *y*-axis perpendicular to the participant body on the 2D workspace was kept with the same sign). Once the EMG channels were selected and the features extracted, a myoelectric decoder was calibrated for each DoF with all the feature set and the corresponding flipped kinematics (linear or angular velocity), and then kept fixed throughout the subsequent closed-loop control sessions. The decoding algorithm was a ridge regression, which has proven to outperform other linear algorithms for myoelectric applications (Sarasola-Sanz et al., 2015). The regularization parameter λ was fixed at 10^4^, a value that was empirically found to attain a good bias-variance tradeoff.

### Myoelectric Control Paradigm

During the myoelectric control, the features extracted from the produced EMG in real-time were normalized based on the previous minute of data and fed to the myoelectric decoder, which predicted the velocity of each DoF [*V*_*EMG*_of Equation (1) below]. This output velocity was smoothed with a recursive moving average filter that contained the last ten outputs and gave more relevance to the most recent ones (i.e., linearly increasing weights from the past to the most recent outputs). This filtering step ensured a smoother control of the exoskeleton and prevented unwanted jerky movements due to noise or non-stationarities in the EMG signal.

We implemented a partially assisted control scheme to avoid initial frustration due to the complexity of the task. Hence, the velocity for each DoF that was sent to the exoskeleton and that described its movement during the trial periods was determined by the following equation (see Figure 1B):


(1) $$V_{n\,e\,t}=\left(1-\gamma \right)\cdot V_{a\,s\,s\,i\,s\,t}+\gamma \cdot V_{E\,M\,G}$$

Where *V*_*net*_ is the velocity sent to each DoF of the exoskeleton; *V*_*assist*_ is the assistive component that redirects the exoskeleton toward the target [computed with a Linear Quadratic Regulator (LQR) (Shanechi et al., 2016)], and hence, partially corrects any possible deviation due to an erroneous EMG control; *V*_*EMG*_ is the velocity predicted by the mirror decoder from the EMG activity exerted by the left arm; and γ∈ [0, 1] is the weight determining the influence of each component on the net velocity command sent to the exoskeleton (e.g., γ = 0.7 during the trials with 30% of assistance). Therefore, the closed-loop control was established by linking the movement volition detected from the EMG signals with the actual movement of the exoskeleton attached to their left limb. Subjects were continuously provided with information about their EMG muscle activations in the form of velocity modulation. Hence, they had to understand and learn the mapping between their EMG activity and the changes in the trajectory and speed of the movement. Based on this visual and proprioceptive feedback, they had to produce EMG patterns similar to those encoded in the reference model, mirrored from the opposite arm, in order to bring the exoskeleton toward the target position as quickly and as smoothly as possible.

The calibration and reference blocks were fully assisted (γ = 1). As in the TBs, the LQR control theory framework was used for the trajectory estimation in the CB and RB blocks. The average trial time was 13.5 ± 1.1 s, depending on the distance to the target defined for each participant, according to their range of motion.

### Performance Metrics

The following five metrics were selected to evaluate different aspects of the EMG-decoding and of the participants’ performance during the myoelectric control:

• Execution time (percentage): represents the percentage of the total time allowed per movement (i.e., 30 s) that the participants took to reach the target.
• Timeouts (percentage): represents the percentage of trials that were not accomplished because the participants ran out of time before they reached the target.
• Spectral Arc Length (SPARC): measures the smoothness of the movement in each DoF, being more reliable and less noise-sensitive than other commonly used path smoothness metrics (Balasubramanian et al., 2015); the closer to zero the SPARC value, the smoother the trajectory.
• Correlation coefficient (CC): reflects the coherence of the assistive (*V*_*assist*_) and the EMG (*V*_*EMG*_) velocity components that are summed to send a net command to the exoskeleton.
• Normalized root-mean-square-error (NRMSE): measures the difference between the assistive velocity component (*V*_*assist*_) and the EMG-based predicted velocity component (*V*_*EMG*_).

Although SPARC, CC, and NRMSE were computed for each DoF individually, they were analyzed in three different ways, i.e., by averaging: (1) across all the DoFs (*_all); (2) across the DoFs of the upper arm and forearm only (*_proximal); and (3) across the DoFs of the wrist and the hand only (*_distal). Assessing the performance of the proximal and the distal DoFs separately allowed us to investigate whether the fact that the task completion was dependent only on the proximal DoFs influenced the learning process and the control over the different segments of the arm, i.e., reinforcement and instrumental learning effects.

The first three metrics (i.e., Execution time, Timeouts, and SPARC) are behavioral metrics and represent the ability of the participants to control the exoskeleton with their EMG activity, while whereas we considered the last two to be electrophysiological metrics (i.e., CC and NRMSE) that measure the ability of the subjects to match the movement template of the assistive control via the EMG decoder (i.e., to modulate their EMG activity patterns to produce kinematics that are aligned with the assistive component). Note that since the movements during the RBs were fully-assisted and predefined (i.e., the myoelectric control was not active), the participants did not have any influence on the execution time nor on the path smoothness and so, the first three metrics were not computed for this type of blocks.

### Analyses

Using the aforementioned metrics, we performed four analyses to study the effectiveness, usability and acceptability of the system:

#### Motor Control

We hypothesized that the subjects would be able to learn the EMG-to-kinematics model and achieve a more dexterous control of the exoskeleton, reflected in a positive evolution of the performance over time. During the TBs, we could analyze the online muscle activity adaptation to the exoskeleton motor control, which would reflect sensorimotor adaptation based on afferent information (visual, proprioceptive, and haptic) about the ability to reach the target. On the other hand, during RBs we could analyze the generalization and retention of the newly learned EMG-to-kinematics map during the training, as participants were instructed to actively follow the exoskeleton movements even if their EMG activity had no influence on the exoskeleton kinematics during these trials. We therefore studied the motor control performance across and within sessions separately for TBs and RBs.

For the across session analysis, the performance values achieved in the TBs or RBs of the four closed-loop control sessions were concatenated, and a multivariate linear model was fitted with two variables: one indicating the block number (TBs: 1–20; RBs: 1–8), and the other one the session number. The intercept determined the initial performance and the slope defined the learning rate. Positive slope values indicate an increase in performance over time for all the metrics except for the NRMSE, for which it means a decrease.

Similarly, we assessed motor control within sessions. We averaged the performance of each TB or RB across the closed-loop control sessions (i.e., averaging all the first blocks of the four sessions, all the second blocks, etc.). The evolution of the performance during the TBs was modeled with a first order univariate polynomial, where the variable represented the block number (1–5). The mean performance of the two RBs within session were compared with a Wilcoxon non-parametric test.

#### Perception

In this analysis, we studied the correlation between the ratings of the subjects about the difficulty of each trial and their ability to control the exoskeleton during that trial (i.e., with the first three performance metrics: Execution Time, Timeouts, and SPARC). Thus, for each subject, we computed the three performance metrics on a trial-by-trial basis and looked for a correlation with the corresponding ratings (Kendall tau correlation). Finally, after checking for normality, we applied a one-way repeated measures analysis of variance (ANOVA) to compare the correlation values across all the performance metrics and hence, evaluated which metric was perceived as a better indicator of performance by the participants.

In addition, we investigated whether the participants perceived the different assistance levels, despite the fact that they were not informed about their existence. We computed the mean rating of all the trials with the same assistance level (low = 10%, medium = 30%, or high = 60%) for each session and compared these values with a two-way repeated measures ANOVA [Factors: assistance level (low, medium, and high) and session (S2–S5)]. We performed *post hoc* pairwise comparisons between the significant sub-factors and controlled for multiple comparisons using the Bonferroni correction.

#### Assistance Level – Performance Relationship

We evaluated the variability of the performance as a function of the assistance level. We expected that a higher level of assistance would facilitate the control of the exoskeleton, which would be reflected in a shorter execution time and a smoother trajectory. We used the first three metrics (i.e., Execution time, Timeouts, and SPARC) to measure the ability of the subject to control the exoskeleton during each closed-loop control session (S2–S5) and for each assistance level (i.e., low = 10%, medium = 30%, and high assistance = 60%). We compared these values with a two-way repeated measures ANOVA [Factors: assistance level (low, medium, and high) and session (S2–S5)]. We performed *post hoc* pairwise comparisons and corrected them using the Bonferroni method.

#### Feedback Questionnaire

The responses to the feedback questionnaire (see questionnaire template in Supplementary Material) were numeric, on a range from 0 (most negative value) to 10 (most positive value). In order to simplify the analysis, the questions were classified into the following groups:

• Exoskeleton functioning: evaluated whether the exoskeleton moved smoothly and at a comfortable speed.
• Exoskeleton hardware: comprised questions about how comfortable it was to wear and operate the exoskeleton.
• Ease of controlling the exoskeleton (subdivided into proximal and distal DoFs): participants were asked to rate how difficult it was for them to control the movement of the exoskeleton over the proximal and distal DoFs with their EMG activity.
• Feedback accuracy: evaluated the perception of the participants about the feedback provided (i.e., whether they felt that the exoskeleton assisted the movement or instead, it moved against their will).
• Protocol design, pauses and rest periods: looked for the opinion of the participants regarding how tired they were after the training, whether the pauses were long enough, etc.

The average of the responses of all the participants was study their general perception about the listed features of the system and the experimental protocol.

## Results

All participants could control the seven-DoFs of the exoskeleton in real-time with the muscle activity of their left arm (see Supplementary Video 1), with a total of 92.2% of movements accomplished by all the participants within the 30-s time limit.

### Motor Control

We evaluated motor control and investigated the occurrence of a learning process both across and within sessions. These two analyses were applied separately to the TBs and the RBs.

**FIGURE 2:**
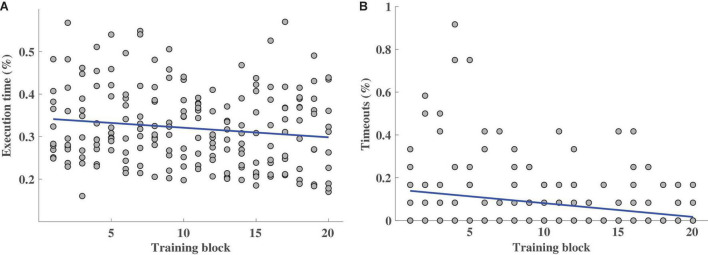
Performance measured by Execution time **(A)** and Timeouts **(B)** metrics for the 20 training blocks from the closed-loop control session (TB1–TB5 of S2–S5), for all the participants. Each circle stands for the mean value (in the [0, 1] range) of all the trials per block and per participant. The polynomial model fitted to the outcome values (in blue) represents the performance trend.

The results of the analysis of the TBs across sessions are illustrated in Figure 2. The linear model fitted to those values exhibits a significant negative slope for Execution time (*p* = 0.041, slope = −0.0023, Figure 2A) and Timeouts (*p* = 3.35⋅10^−4^, slope = −0.0064, Figure 2B). These negative correlations demonstrate improved motor control characterized by shorter time periods needed to reach the target as well as fewer failures to reach the target over the course of the experiment. Some participants produced progressively smoother paths over sessions (see Supplementary Figure 1), although the tendency was not significant when looking at all the subjects together (see Supplementary Figures 2A,B). The CC and NRMSE values in Supplementary Figures 1C–F show that the control of the distal DoFs was poorer toward the end, as reflected by the significant (*p* = 0.032, slope = 8.81⋅10^−4^) positive slope of NRMSE values.

However, this did not happen for the proximal DoFs, noted that the ability of the participants to adapt the EMG activity was significantly (*p*_*CC*_ = 0.0098, slope = 7.60⋅10^−4^; *p*_*NRMSE*_ = 0.002, slope = −1.28⋅10^−4^) higher for the proximal DoFs than for the distal DoFs during the closed-loop myoelectric control (mean *CC*_*proximal*_ = 0.355 ± 0.175; mean *CC*_*distal*_ = 0.141 ± 0.254; mean *NRMSE*_*proximal*_ = 0.164 ± 0.020; mean *NRMSE*_*distal*_ = 0.199 ± 0.028). In the within-session analysis, there were no learning trends or significant performance improvements for any of the metrics (see Supplementary Figure 3).

### Perception

**FIGURE 3:**
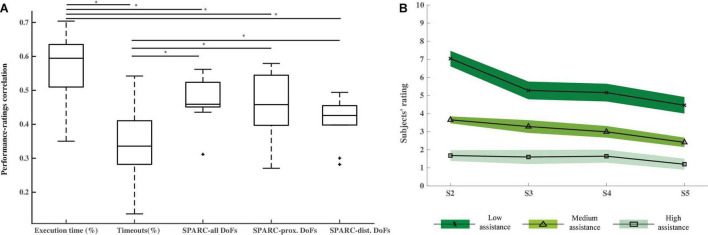
Perception. **(A)** Kendall tau correlations (including standard deviation and median) between the difficulty ratings given by the participants after each trial and the performance measured by the Execution time, Timeouts, and SPARC metrics. Asterisks show significant differences (*p* < 0.05) and “+” symbols are outliers. **(B)** Mean and standard error of the difficulty ratings given by all the participants after each trial for each closed-loop control session (S2–S5) and computed separately for each assistance level. The higher the rating, the more difficult was for the participants to control the exoskeleton. Corrected pairwise comparisons show significant differences between the ratings given to the three assistance level trials.

The correlation between the performance and the ratings given by the participants lay within the [0.1, 0.7] range, as can be observed in Figure 3A. The ANOVA shows a significant (*p* < 10^−6^) difference between the correlation values of the metrics. Execution time happened to be the most intuitive metric for the participants to rate the difficulty of the trial, reflected in significantly higher mean correlation values (Execution time vs. Timeouts: *p* = 0.024; vs. SPARC–all: *p* = 1⋅10^−6^; vs. SPARC-proximal: *p* = 4⋅10^−6^; vs. SPARC – distal: *p* = 1⋅10^−6^) whereas Timeouts was the metric with the lowest correlation values (Timeouts vs. Execution time: *p* = 0.024; vs. SPARC–all: *p* = 3⋅10^−6^; vs. SPARC-proximal: *p* = 6⋅10^−6^; vs. SPARC – distal: *p* = 3⋅10^−6^).

The mean ratings of the low, medium and high assistance level trials across all the participants for each session are presented in Figure 3B. The two-way ANOVA applied to these values confirms that the participants could perceive the different assistance levels, as reflected by the significantly different ratings given to the low, medium and high assistance trials (corrected pairwise comparisons: low vs. high: *p* = 2.2⋅10^−5^; low vs. medium: *p* = 1.2⋅10^−4^; medium vs. high: *p* = 3.5 10^−5^). Interestingly, the two-way ANOVA showed a significant (*p* = 0.002) session effect and significant pairwise differences between the ratings of sessions S2 and S5 (*p* = 0.008) and S3 and S5 (*p* = 0.019), meaning that participants found the various assistance level trials less difficult over sessions.

### Assistance Level – Performance Relationship

**FIGURE 4:**
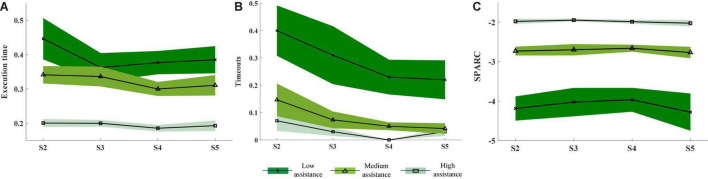
Mean and standard error of the performance measured by the Execution time **(A)**, Timeouts **(B)**, and SPARC **(C)** for the different assistance levels (high: light green and squares; medium: medium green and triangles; low: dark green and crosses). The performance values for all the participants were averaged for each closed-loop control session (S2–S5).

As can be seen in Figure 4, higher assistance values led to higher performance. Indeed, Assistance level had a significant effect on all the metrics, as reflected by the two-way ANOVA (Execution time: *p* = 1.1 10^−5^; Timeouts: *p* = 4.9 10^−5^; SPARC: *p* < 10^−6^). The *post hoc* comparisons show significant differences between the three assistance levels, except for the low vs. medium levels of Execution time and the medium vs. high level of Timeouts. A decrease of the timeouts over sessions for all the three assistance levels can also be noticed and was reflected by a significant (*p* = 0.015) effect of the session factor in the two-way ANOVA, although *post hoc* comparisons did not show any pairwise differences after Bonferroni correction.

### Feedback Questionnaire

**FIGURE 5:**
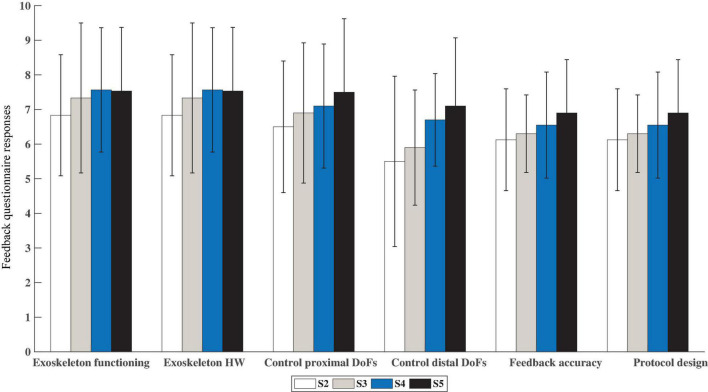
Mean and standard deviation of the responses given by the participants to the feedback questionnaire after each closed-loop control session (S2–S5). The questions were grouped into five different categories: Exoskeleton functioning, Exoskeleton Hardware, Control of the exoskeleton (proximal and distal DoFs), Feedback accuracy and Protocol design. Higher values mean more positive feedback (Max = 10 pts).

Figure 5 illustrates the average responses of the participants to the questionnaires filled out at the end of each closed-loop control session. It shows satisfactory response values (in the range [6.3, 8.0]) that remained stable across sessions for the questions related to the exoskeleton functioning and hardware as well as the protocol design. Additionally, participants reported an increasing ease in controlling the exoskeleton over the sessions. From these values, a noticeable difference between the proximal and distal DoFs can be detected too, with the control over the proximal DoFs being apparently more intuitive than the distal DoFs. However, the responses about the control over the distal DoFs show that participants also found the control of those DoFs easier toward the later sessions. The reason for this might be that participants were not told that the trial accomplishment depended only on the position of the proximal DoFs. Thus, they probably associated the reduction in execution time and number of timeouts over sessions with a better control of both proximal and distal DoFs. Finally, the ratings for the feedback accuracy were slightly more positive over sessions with mean values ranging from 6.1 to 6.9.

## Discussion

In this study, we presented and validated a myoelectric interface intended for the upper limb rehabilitation of stroke patients. The system includes a ridge regression algorithm, a subject-specific myoelectric mirror model, fixed within and across sessions, and high-resolution EMG recordings.

This is the first myoelectric system that has been successfully used to simultaneously control a seven-DoF exoskeleton in real-time, including proximal and distal joints. It has previously been demonstrated that training functional tasks, involving coordinated proximal and distal joint movements of the arm, might facilitate the activation of more affected distal muscles in patients with motor impairment, and ease the transfer of the acquired skills to activities of daily living (Takeuchi and Izumi, 2012; Garcia-Cossio et al., 2013). However, the existing non-invasive myoelectric systems do not offer the possibility of training coordinated multi-joint tasks, since they are restricted to the real-time simultaneous and proportional control of up to four DoFs (proximal or distal) of an external robot (de Rugy et al., 2013; Pistohl et al., 2013; Ison and Artemiadis, 2015; Ison et al., 2016), two DoFs of a prosthesis (Fougner et al., 2014; Amsuess et al., 2016) or up to five DoFs of an upper-limb exoskeleton for up to three pairs of elbow or hand movement classification (Tang et al., 2014; Lu et al., 2017). The difficulty of continuously and accurately predicting users’ motion intention from EMG signals and of simultaneously controlling the velocity of several DoFs of an exoskeleton in real-time has limited its clinical and commercial use. This study goes a step further and addresses the challenge of integrating the visual and proprioceptive feedback provided by a multi-DoF exoskeleton, which constitutes a key feature of rehabilitation therapies and is a rather difficult task than controlling an external robot or a simple visual artifact. Thus, in this exploratory study, we evaluated if the provided feedback and assistance levels would feel natural for the users and were good enough to learn the mapping and achieve successful control of the multi-DoF interface over time. The results prove that our mirror myoelectric system allows safe, smooth and continuous control of a seven DoF exoskeleton in real-time.

This study was limited to the training of a predefined set of movements and target positions. Despite there were no space restrictions other than the size of the workspace, participants were explicitly asked to avoid exploratory free movements and to reach the targets following the shortest and fastest possible trajectory, as shown in the reference blocks at the beginning of each session. In a longitudinal intervention with patients, allowing them to explore different strategies would be interesting and parameters such as the target positions, range of motion, assistance level and tasks could be adapted according to their evolution. Hence, this is an uncontrolled study limited to specific conditions, as we were seeking to initially prove the safety, usability and effectiveness of our system in healthy participants. In this initial validation, we demonstrated that this mirror interface and training strategy can induce learning of a mapping based on natural EMG patterns (independent of what EMG patterns the other arm had, i.e., ignoring interlimb differences) and so, might be a valid method to elicit relearning of healthy EMG patterns during functional task training in stroke patients. Nevertheless, this remains to be tested in a controlled clinical trial.

The results of the analysis across sessions demonstrate that the participants learned the mirror mapping and operated the myoelectric interface in a progressively more efficient way over the training period (see Figure 2). The kinematics predicted from the EMG activity defined a more direct and accurate movement toward the aimed position, reflected in higher speeds and in turn, lower time periods needed to reach a target. This was confirmed by the progressively more positive responses in subjects’ responses to the feedback questionnaire regarding feedback accuracy and ease in controlling the exoskeleton (see Figure 5) and the lower difficulty ratings over sessions (see Figure 3B). The performance also varied according to the assistance level. As expected, the higher the assistance, the shorter the execution time, the smaller the percentage of timeouts and the smoother the path. This difference could also be perceived by the participants, as revealed by the significantly different ratings given for the trials of each assistance level. More importantly, it was demonstrated that even during the trials with only 10% assistance, participants could successfully control the velocity of a seven-DoF exoskeleton with their EMG during a complex functional task. Indeed, they produced significantly (*p* = 0.015) fewer timeouts during the trials of any of the three assistance levels over the course of the experiment (see Figure 4B), and reported progressively less difficulty (*p* = 0.002) to perform the trials with any assistance level (see Figure 3B). Furthermore, the participants rated satisfactorily the usability and comfort of the system and provided us with useful information to adapt and optimize the system for future experiments. Therefore, the results are encouraging and have relevant implications for its future application to a rehabilitation scenario with stroke patients.

There is a trend toward a degradation of the control (i.e., smaller CC and larger NRMSE values) of the distal DoFs over the course of the intervention (see Figure 2). Conversely, performance of the proximal DoFs improved over time. This difference goes in line with the results found in previous offline studies in healthy and stroke patients (Sarasola-Sanz et al., 2015, 2017). One reason for the difference between the proximal and distal DoFs performance could be that the movements of the distal DoFs were finer and more difficult to control than the proximal DoFs, as reported by the participants in the feedback questionnaire and by the significantly (*p*_*CC*_ = 0.0098; *p*_*NRMSE*_ = 0.002) lower mean performance of the distal DoFs compared to the proximal ones. In addition, the fact that the task completion condition was based only on the proximal DoF position could have influenced the performance. Despite the fact that participants could control and receive feedback on the distal DoFs, they were never informed whether the target position was successfully reached on those DoFs or not. This lack of information might have impeded the occurrence of a learning process (based on reinforcement learning, i.e., on reward), reflected in a more accurate control over the distal DoFs. This supports the importance of receiving contingent sensory feedback (i.e., exoskeleton moving according to muscle activity) and reward (i.e., beep indicating task completion) of the DoFs being controlled for motor control (Todorov and Jordan, 2002; Scott, 2004; Ramos-Murguialday et al., 2013). Furthermore, there is a significant increase in variability in CC and NRMSE values in the distal DoFs during RBs (see Supplementary Figures 3B,D), which suggests implicit motor exploration or different motor control strategies.

The concepts of skill acquisition and motor adaptation and their relationship with motor recovery is still unclear (Kitago and Krakauer, 2013). Dipietro et al. (2012) suggested that an internal map of the trained task is built by the brain during motor recovery, following a process more similar to motor skill acquisition than motor adaptation. Others have stated that repeated adaptation can lead to learning a new and more permanent motor skill that can cause long-lasting changes in the motor cortex and the cerebellum (Bastian, 2008). The results of our study demonstrate that four training sessions sufficed for healthy individuals to learn the imposed mapping and achieve proficient myoelectric control. However, from these results we cannot conclude whether the observed learning was the result of an adaptation or a skill acquisition process. The metrics assessing the myoelectric control (i.e., CC and NRMSE) during the RBs partially reflect the observed significant behavioral changes, indicating an intrinsically effective generalization and retention of the imposed mirror EMG-to-kinematics map. This is of great importance as this might open a new door to efficient re-learning of correct EMG activation patterns. However, our results were not significant and in the distal DoFs, which were not rewarded during the training, the effects were mixed and inconclusive. A larger number of participants or sessions might be needed to find significant effects during the training.

It should also be noticed that some of the participants may have partially or completely relied on strategies such as the reduction of interaction forces to accomplish the trials in a shorter time over blocks. The absence of sensors to measure such parameters is a limitation of the study that will be addressed in future experiments. The introduced assistance levels also helped the participants to navigate toward the target and some participants might have relied on this component and completely relaxed their muscles. However, the introduced assistance components were not big enough to reach the target within the timeout time in the absence of voluntary EMG contractions and the experimenter controlled that the subjects actively tried to bring the exoskeleton toward the target at all times. In a rehabilitation scenario, one could rely on the active involvement of participants since the outcome greatly depends on their engagement. Alternatively, a movement detection (e.g., rest vs. movement-decoder gating the exoskeleton continuous EMG control) could be implemented in order to always ensure a voluntary activation. Additionally, it would also be necessary to assess the generalization of the gains to untrained tasks and the long-term effects of the training in order to determine which specific motor learning process occurred during the intervention. Nevertheless, the observed multi-DoF myoelectric control and positive feedback from the healthy population encourage the transfer of this rehabilitation system to the clinical stage.

This mirror decoder has been designed for the rehabilitation of stroke patients, as they typically keep the motor abilities of one of their limbs intact or mostly intact. The system takes advantage of this characteristic by using the muscle activation patterns of their intact limb as a reference mapping for patients to learn to move their paretic limb through the correct recruitment of their muscles. Higher decoding accuracies could probably be achieved when building the decoder by correlating EMG activity of their paretic arm with the intended movement. However, training with such decoder could indeed reinforce the pathological patterns of their paretic limb avoiding the relearning of healthy activity and thus, rehabilitation (Cesqui et al., 2013). It should also be noticed that using a mapping built with EMG of their intact limb avoids generalization issues derived from the use of decoders built with other healthy individuals’ EMG activity, who present different neurophysiological characteristics (age, sex, arm size, strength, etc.) (Sarasola-Sanz et al., 2018).

Previous evidence suggests that the learning of a new neuromotor mapping is associated with the emergence of new muscle activation patterns or synergies. Moreover, these effects can persist after a week facilitating the generalization to new tasks while keeping the same mapping (Ison and Artemiadis, 2015). Other studies have also emphasized the importance of error strategies, guidance and feedback for muscle activation modulation and motor learning (Patton et al., 2006; Emken et al., 2007; Sigrist et al., 2015; Marchal-Crespo et al., 2017). They demonstrated that guidance and feedback improve motor task learning and that errors should not be reduced or eliminated to induce learning, but instead they should be shown and in some cases, even amplified (Marchal-Crespo et al., 2017). Therefore, based on the results, we believe that training with this closed-loop system that provides the necessary assistance and the appropriate response stimuli (i.e., contingent feedback about the paretic EMG activity), stroke patients will be able to learn the fixed mapping of healthy activity imposed by the mirror decoder. Whether this learning process leads to the formation of new and healthy muscle synergies in the paretic arm and consequently to motor rehabilitation, remains to be investigated in the stroke population.

Many patients classified by standard scales as having completely paralyzed joints (i.e., no visually perceived movement) nevertheless retain significant residual muscle activity, which could be decoded and used to control external devices (Ramos-Murguialday et al., 2015). One of the limitations of the system presented here is that patients with no residual muscle activity could not benefit from it. However, patients without decodable muscle activity could initially train with brain-machine-interfaces (BMIs) until they recover enough EMG activity (Ramos-Murguialday et al., 2013; Balasubramanian et al., 2018) to profit from myoelectric interfaces or from hybrid-BMIs, with a shared brain and muscle control (Sarasola-Sanz et al., 2017). Despite its limitations, our approach opens the doors of rehabilitation to many stroke patients who retain minimal EMG activity but cannot benefit from other therapies such as constraint induced movement therapy and bilateral arm training (Birbaumer et al., 2008) that require residual movement of the paretic limb.

## Data Availability Statement

The raw data supporting the conclusions of this article will be made available by the authors, without undue reservation.

## Ethics Statement

The studies involving human participants were reviewed and approved by the Faculty of Medicine of the University of Tübingen, Germany. The patients/participants provided their written informed consent to participate in this study.

## Author Contributions

AS-S, EL-L, NI-L, JM, and AR-M conceived and designed the experiments and manuscript preparation. AS-S, EL-L, NI-L, GR, and TF performed the experiments. AS-S, EL-L, GR, TF, and AR-M analyzed the data. All authors contributed to the article and approved the submitted version.

## Conflict of Interest

The authors declare that the research was conducted in the absence of any commercial or financial relationships that could be construed as a potential conflict of interest.

## Publisher’s Note

All claims expressed in this article are solely those of the authors and do not necessarily represent those of their affiliated organizations, or those of the publisher, the editors and the reviewers. Any product that may be evaluated in this article, or claim that may be made by its manufacturer, is not guaranteed or endorsed by the publisher.

## References

[B1] Amsuess S. Vujaklija I. Goebel P. Roche A. D. Graimann B. Aszmann O. C. (2016). Context-dependent upper limb prosthesis control for natural and robust use. *IEEE Trans. Neural. Syst. Rehabil. Eng.* 24 744–753. 10.1109/TNSRE.2015.2454240 26173217

[B2] Antuvan C. W. Ison M. Artemiadis P. (2014). Embedded human control of robots using myoelectric interfaces. *IEEE Trans. Neural. Syst. Rehabil. Eng.* 22 820–827. 10.1109/TNSRE.2014.2302212 24760930

[B3] Arya K. N. Pandian S. Verma R. Garg R. K. (2011). Movement therapy induced neural reorganization and motor recovery in stroke: a review. *J. Bodyw. Mov. Ther.* 15 528–537. 10.1016/J.JBMT.2011.01.023 21943628

[B4] Balasubramanian S. Garcia-Cossio E. Birbaumer N. Burdet E. Ramos-Murguialday A. (2018). Is EMG a viable alternative to bci for detecting movement intention in severe stroke? *IEEE Trans. Biomed. Eng.* 65 2790–2797. 10.1109/TBME.2018.2817688 29993449

[B5] Balasubramanian S. Melendez-Calderon A. Roby-Brami A. Burdet E. (2015). On the analysis of movement smoothness. *J. Neuroeng. Rehabil.* 12:112. 10.1186/s12984-015-0090-9 26651329 PMC4674971

[B6] Bastian A. J. (2008). Understanding sensorimotor adaptation and learning for rehabilitation. *Curr. Opin. Neurol.* 21 628–633. 10.1097/WCO.0b013e328315a293 18989103 PMC2954436

[B7] Birbaumer N. Murguialday A. R. Cohen L. (2008). Brain-computer interface in paralysis. *Curr. Opin. Neurol.* 21 634–638. 10.1097/WCO.0b013e328315ee2d 18989104

[B8] Bizzi E. Cheung V. C. (2013). The neural origin of muscle synergies. *Front. Comput. Neurosci.* 7:51. 10.3389/fncom.2013.00051 23641212 PMC3638124

[B9] Bizzi E. Cheung V. C. d’Avella A. Saltiel P. Tresch M. (2008). Combining modules for movement. *Brain Res. Rev.* 57 125–133. 10.1016/J.BRAINRESREV.2007.08.004 18029291 PMC4295773

[B10] Cesqui B. Tropea P. Micera S. Krebs H. (2013). EMG-based pattern recognition approach in post stroke robot-aided rehabilitation: a feasibility study. *J. Neuroeng. Rehabil.* 10:75. 10.1186/1743-0003-10-75 23855907 PMC3729537

[B11] Chan D. Y. Chan C. C. Au D. K. (2006). Motor relearning programme for stroke patients: a randomized controlled trial. *Clin. Rehabil.* 20 191–200. 10.1191/0269215506cr930oa 16634338

[B12] Cramer S. C. Parrish T. B. Levy R. M. Stebbins G. T. Ruland S. D. Lowry D. W. (2007). Predicting functional gains in a stroke trial. *Stroke* 38 2108–2114. 10.1161/STROKEAHA.107.485631 17540966

[B13] d’Avella A. Saltiel P. Bizzi E. (2003). Combinations of muscle synergies in the construction of a natural motor behavior. *Nat. Neurosci.* 6 300–308. 10.1038/nn1010 12563264

[B14] de Rugy A. D. Loeb G. E. Carroll T. J. (2013). Are Muscle Synergies Useful for Neural Control? *Front. Comput. Neurosci.* 7:19. 10.3389/fncom.2013.00019 23519326 PMC3604633

[B15] Dipietro L. Krebs H. I. Volpe B. T. Stein J. Bever C. Mernoff S. T. (2012). Learning, not adaptation, characterizes stroke motor recovery: evidence from kinematic changes induced by robot-assisted therapy in trained and untrained task in the same workspace. *IEEE Trans. Neural. Syst. Rehabil. Eng.* 20 48–57. 10.1109/TNSRE.2011.2175008 22186963 PMC4687974

[B16] Emken J. L. Benitez R. Reinkensmeyer D. J. (2007). Human-robot cooperative movement training: learning a novel sensory motor transformation during walking with robotic assistance-as-needed. *J. Neuroeng. Rehabil.* 4:8. 10.1186/1743-0003-4-8 17391527 PMC1847825

[B17] Fougner A. L. Stavdahl O. Kyberd P. J. (2014). System training and assessment in simultaneous proportional myoelectric prosthesis control. *J. Neuroeng. Rehabil.* 11:75. 10.1186/1743-0003-11-75 24775602 PMC4041142

[B18] Garcia-Cossio E. Birbaumer N. Ramos-Murguialday A. (2013). “Facilitation of completely paralyzed forearm muscle activity in chronic stroke patients,” in *Proceeding of the 2013 6th International IEEE/EMBS Conference on Neural Engineering (NER)* (San Diego, CA: IEEE), 1545–1548. 10.1109/NER.2013.6696241

[B19] Hosp J. A. Luft A. R. (2011). Cortical plasticity during motor learning and recovery after ischemic stroke. *Neural Plast.* 2011:871296. 10.1155/2011/871296 22135758 PMC3202122

[B20] Ison M. Antuvan C. W. Artemiadis P. (2014b). “Learning Efficient Control of Robots Using Myoelectric Interfaces,” in *Proceeding of the 2014 IEEE International Conference on Robotics and Automation (ICRA)* (Hong Kong: IEEE), 2880–2885. 10.1109/ICRA.2014.6907273

[B21] Ison M. Artemiadis P. (2015). Proportional myoelectric control of robots: muscle synergy development drives performance enhancement. *Retainment Generalization IEEE Trans. Rob.* 31 259–268. 10.1109/TRO.2015.2395731

[B22] Ison M. Artemiadis P. Gillis L. Heard D. C. Donovan W. H. Atkins D. J. (2014a). The role of muscle synergies in myoelectric control: trends and challenges for simultaneous multifunction control. *J. Neural Eng.* 11:051001. 10.1088/1741-2560/11/5/051001 25188509

[B23] Ison M. Vujaklija I. Whitsell B. Farina D. Artemiadis P. (2016). High-density electromyography and motor skill learning for robust long-term control of a 7-dof robot arm. *IEEE Trans. Neural. Syst. Rehabil. Eng.* 24 424–433. 10.1109/TNSRE.2015.2417775 25838524

[B24] Itou T. Terao M. Nagata J. Yoshida M. (2001). “Mouse Cursor Control System Using EMG,” in *In 2001 Conference Proceedings of the 23rd Annual International Conference of the IEEE Engineering in Medicine and Biology Society* (Istanbul: IEEE), 1368–1369. 10.1109/IEMBS.2001.1020453

[B25] Kitago T. Krakauer J. W. (2013). Motor learning principles for neurorehabilitation. *Handb. Clin. Neurol.* 110 93–103. 23312633 10.1016/B978-0-444-52901-5.00008-3

[B26] Kleim J. A. Jones T. A. (2008). Principles of experience-dependent neural plasticity: implications for rehabilitation after brain damage. *J. Speech Lang. Hear. Res.* 51 S225–S239. 10.1044/1092-4388(2008/018 18230848

[B27] Krakauer J. W. (2006). Motor learning: its relevance to stroke recovery and neurorehabilitation. *Curr. Opin. Neurol.* 19 84–90. 10.1097/01.wco.0000200544.29915.cc 16415682

[B28] Krakauer J. W. (2015). “The applicability of motor learning to neurorehabilitation,” in *Oxford Textbook Of Neurorehabilitation*, eds Dietz In V. Ward N. S. (Oxford: Oxford University Press), 55–63. 10.1093/med/9780199673711.003.0007

[B29] Krakauer J. W. Mazzoni P. (2011). Human sensorimotor learning: adaptation, skill, and beyond. *Curr. Opin. Neurobiol.* 21 636–644. 10.1016/J.CONB.2011.06.012 21764294

[B30] Kwakkel G. Wagenaar R. C. Koelman T. W. Lankhorst G. J. Koetsier J. C. (1997). Effects of intensity of rehabilitation after stroke. *Stroke* 28 1550–1556. 10.1161/01.str.28.8.1550 9259747

[B31] Lu Z. Chen X. Zhang X. Tong K-Y Zhou P. (2017). Real-time control of an exoskeleton hand robot with myoelectric pattern recognition. *Int. J. Neural Sys.* 27:1750009. 10.1142/S0129065717500095 27873553

[B32] Marchal-Crespo L. Michels L. Jaeger L. López-Olóriz J. Riener R. (2017). Effect of error augmentation on brain activation and motor learning of a complex locomotor task. *Front. Neurosci.* 11:526. 10.3389/fnins.2017.00526 29021739 PMC5623679

[B33] Marchal-Crespo L. Reinkensmeyer D. J. (2009). Review of control strategies for robotic movement training after neurologic injury. *J. Neuroeng. Rehabil.* 6:20. 10.1186/1743-0003-6-20 19531254 PMC2710333

[B34] Muller-Putz G. Leeb R. Tangermann M. Hohne J. Kubler A. Cincotti F. (2015). Towards noninvasive hybrid brain-computer interfaces: framework. practice, clinical application, and beyond. *Proc. IEEE* 103 926–943. 10.1109/JPROC.2015.2411333

[B35] Patton J. L. Stoykov M. E. Kovic M. Mussa-Ivaldi F. A. (2006). Evaluation of robotic training forces that either enhance or reduce error in chronic hemiparetic stroke survivors. *Exp. Brain Res.* 168 368–383. 10.1007/s00221-005-0097-8 16249912

[B36] Piron L. Turolla A. Agostini M. Zucconi C. S. Ventura L. Tonin P. (2010). Motor learning principles for rehabilitation: a pilot randomized controlled study in poststroke patients. *Neurorehabil. Neural Repair* 24 501–508. 10.1177/1545968310362672 20581337

[B37] Pistohl T. Cipriani C. Jackson A. Nazarpour K. (2013). Abstract and proportional myoelectric control for multi-fingered hand prostheses. *Ann. Biomed. Eng.* 41 2687–2698. 10.1007/s10439-013-0876-5 23934195 PMC3825263

[B38] Prange G. B. Jannink M. J. A. Groothuis-Oudshoorn C. G. M. Hermens H. J. IJzerman M. J. (2006). Systematic review of the effect of robot-aided therapy on recovery of the hemiparetic arm after stroke. *J. Rehabil. Res. Dev.* 43 171–184. 10.1682/JRRD.2005.04.0076 16847784

[B39] Radhakrishnan S. M. Baker S. N. Jackson A. (2008). Learning a novel myoelectric-controlled interface task. *J. Neurophysiol.* 100 2397–2408. 10.1152/jn.90614.2008 18667540 PMC2576223

[B40] Ramos-Murguialday A. Broetz D. Rea M. Läer L. Yilmaz O. Brasil F. L. (2013). Brain-machine-interface in chronic stroke rehabilitation: a controlled study. *Ann. Neurol.* 74 100–108. 10.1002/ana.23879 23494615 PMC3700597

[B41] Ramos-Murguialday A. García-Cossio E. Walter A. Cho W. Broetz D. Bogdan M. (2015). Decoding upper limb residual muscle activity in severe chronic stroke. *Ann. Clin. Transl. Neurol.* 2 1–11. 10.1002/acn3.122 25642429 PMC4301668

[B42] Reinkensmeyer D. J. Burdet E. Casadio M. John W. K. Kwakkel G. Lang C. E. (2016). Computational neurorehabilitation: modeling plasticity and learning to predict recovery. *J. Neuroeng. Rehabil.* 13:42. 10.1186/s12984-016-0148-3 27130577 PMC4851823

[B43] Reinkensmeyer D. J. Emken J. L. Cramer S. C. (2004). Robotics, motor learning, and neurologic recovery. *Ann. Rev. Biomed. Eng.* 6 497–525. 10.1146/annurev.bioeng.6.040803.140223 15255778

[B44] Sarasola-Sanz A. Irastorza-Landa N. López-Larraz E. Bibian C. Helmhold F. Broetz D. (2017). A hybrid-bmi based on eeg and emg activity for the motor rehabilitation of stroke patients. *IEEE. Int. Conf. Rehabil. Robot* 2017 895–900. 10.1109/EMBC.2018.8512711 28813934

[B45] Sarasola-Sanz A. Irastorza-Landa N. López-Larraz E. Shiman F. Spüler M. Birbaumer N. (2018). Design and effectiveness evaluation of mirror myoelectric interfaces: a novel method to restore movement in hemiplegic patients. *Sci. Rep.* 8:16688. 10.1038/s41598-018-34785-x 30420779 PMC6232088

[B46] Sarasola-Sanz A. Irastorza-Landa N. Shiman F. Lopez-Larraz E. Spuler M. Birbaumer N. (2015). “EMG-based multi-joint kinematics decoding for robot-aided rehabilitation therapies,” in *Proceeding of the 2015 IEEE International Conference on Rehabilitation Robotics (ICORR)* (Singapore: IEEE), 229–234. 10.1109/ICORR.2015.7281204

[B47] Sartori M. Durandau G. Došen S. Farina D. (2018). Robust simultaneous myoelectric control of multiple degrees of freedom in wrist-hand prostheses by real-time neuromusculoskeletal modeling. *J. Neural Eng.* 15:066026. 10.1088/1741-2552/aae26b 30229745

[B48] Scheme E. Englehart K. (2011). Electromyogram pattern recognition for control of powered upper-limb prostheses: state of the art and challenges for clinical use. *J. Rehabil. Res. Dev.* 48:643. 10.1682/JRRD.2010.09.0177 21938652

[B49] Scott S. H. (2004). Optimal feedback control and the neural basis of volitional motor control. *Nat. Rev. Neurosci.* 5 532–545. 10.1038/nrn1427 15208695

[B50] Shadmehr R. Wise S. P. (2005). *The Computational Neurobiology of Reaching and Pointing: A Foundation for Motor Learning.* Cambridge, MA: MIT Press.

[B51] Shanechi M. M. Orsborn A. L. Carmena J. M. (2016). Robust brain-machine interface design using optimal feedback control modeling and adaptive point process filtering. *PLoS Comput. Biol.* 12:1004730. 10.1371/journal.pcbi.1004730 27035820 PMC4818102

[B52] Shmuelof L. Krakauer J. W. (2011). Are we ready for a natural history of motor learning? *Neuron* 72 469–476. 10.1016/J.NEURON.2011.10.017 22078506 PMC3389513

[B53] Sigrist R. Rauter G. Marchal-Crespo L. Riener R. Wolf P. (2015). Sonification and haptic feedback in addition to visual feedback enhances complex motor task learning. *Exp. Brain Res.* 233 909–925. 10.1007/s00221-014-4167-7 25511166

[B54] Song R. Tong K.-Y. Hu X. Zhou W. (2013). Myoelectrically controlled wrist robot for stroke rehabilitation. *J. NeuroEng. Rehabil.* 10:1. 10.1186/1743-0003-10-52 23758925 PMC3685570

[B55] Takeuchi N. Izumi S.-I. (2012). Maladaptive plasticity for motor recovery after stroke: mechanisms and approaches. *Neural Plast.* 2012 1–9. 10.1155/2012/359728 22792492 PMC3391905

[B56] Takeuchi N. Izumi S.-I. (2013). Rehabilitation with poststroke motor recovery: a review with a focus on neural plasticity. *Stroke Res. Treat.* 2013:128641. 10.1155/2013/128641 23738231 PMC3659508

[B57] Tang Z. Zhang K. Sun S. Gao Z. Zhang L. Yang Z. (2014). An upper-limb power-assist exoskeleton using proportional myoelectric control. *Sensors* 14 6677–6694. 10.3390/s140406677 24727501 PMC4029719

[B58] Todorov E. Jordan M. I. (2002). Optimal feedback control as a theory of motor coordination. *Nat. Neurosci.* 5 1226–1235. 10.1038/nn963 12404008

[B59] Tresch M. C. Saltiel P. Bizzi E. (1999). The construction of movement by the spinal cord. *Nat. Neurosci.* 2 162–167. 10.1038/5721 10195201

[B60] Vujaklija I. Shalchyan V. Kamavuako E. N. Jiang N. Marateb H. R. Farina D. (2018). Online mapping of emg signals into kinematics by autoencoding. *J. NeuroEng. Rehabil.* 15:21 10.1186/s12984-018-0363-1 29534764 PMC5850983

[B61] Wolpert D. M. Diedrichsen J. Flanagan J. R. (2011). Principles of sensorimotor learning. *Nat. Rev. Neurosci.* 12 739–751. 10.1038/nrn3112 22033537

