# Supplementary material for: Real-Time Control of a Multi-Degree-of-Freedom Mirror Myoelectric Interface During Functional Task Training
Source: Front Neurosci. 2022 Mar 11;16:764936. doi: 10.3389/fnins.2022.764936 (PMC8962619; doi:10.3389/fnins.2022.764936)
Supplement: Supplementary file 1 [file Data_Sheet_1.pdf]

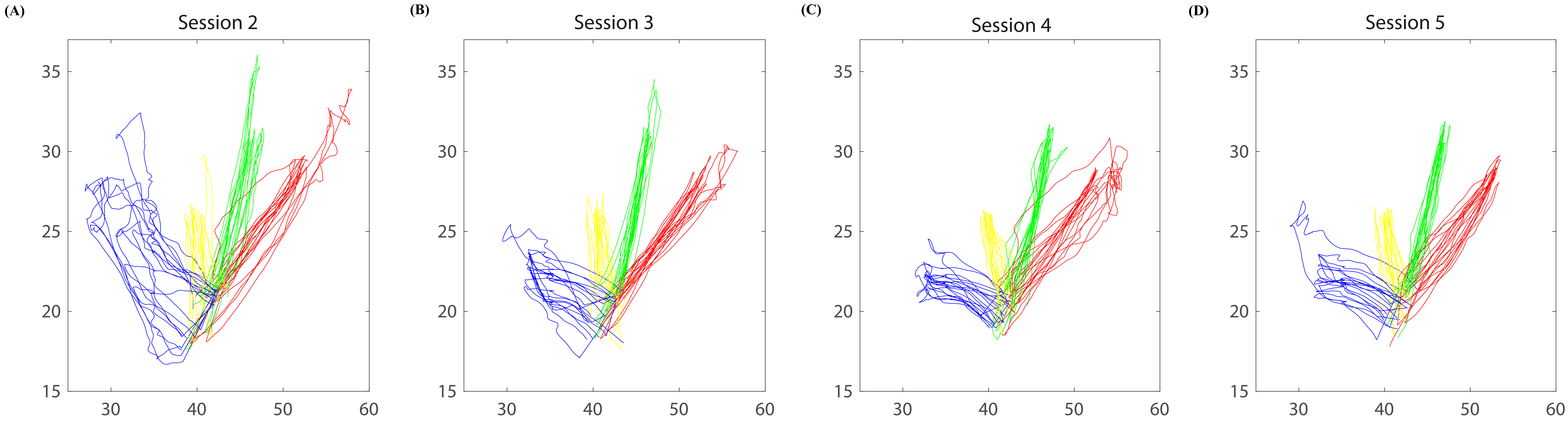

Supplementay Figure 1: Paths followed by one of the participants towards the four target locations (blue, yellow, green and red targets) during the training blocks of sessions 2-5.
