# Supplementary material for: Real-Time Control of a Multi-Degree-of-Freedom Mirror Myoelectric Interface During Functional Task Training
Source: Front Neurosci. 2022 Mar 11;16:764936. doi: 10.3389/fnins.2022.764936 (PMC8962619; doi:10.3389/fnins.2022.764936)
Supplement: Supplementary file 2 [file Data_Sheet_2.pdf]

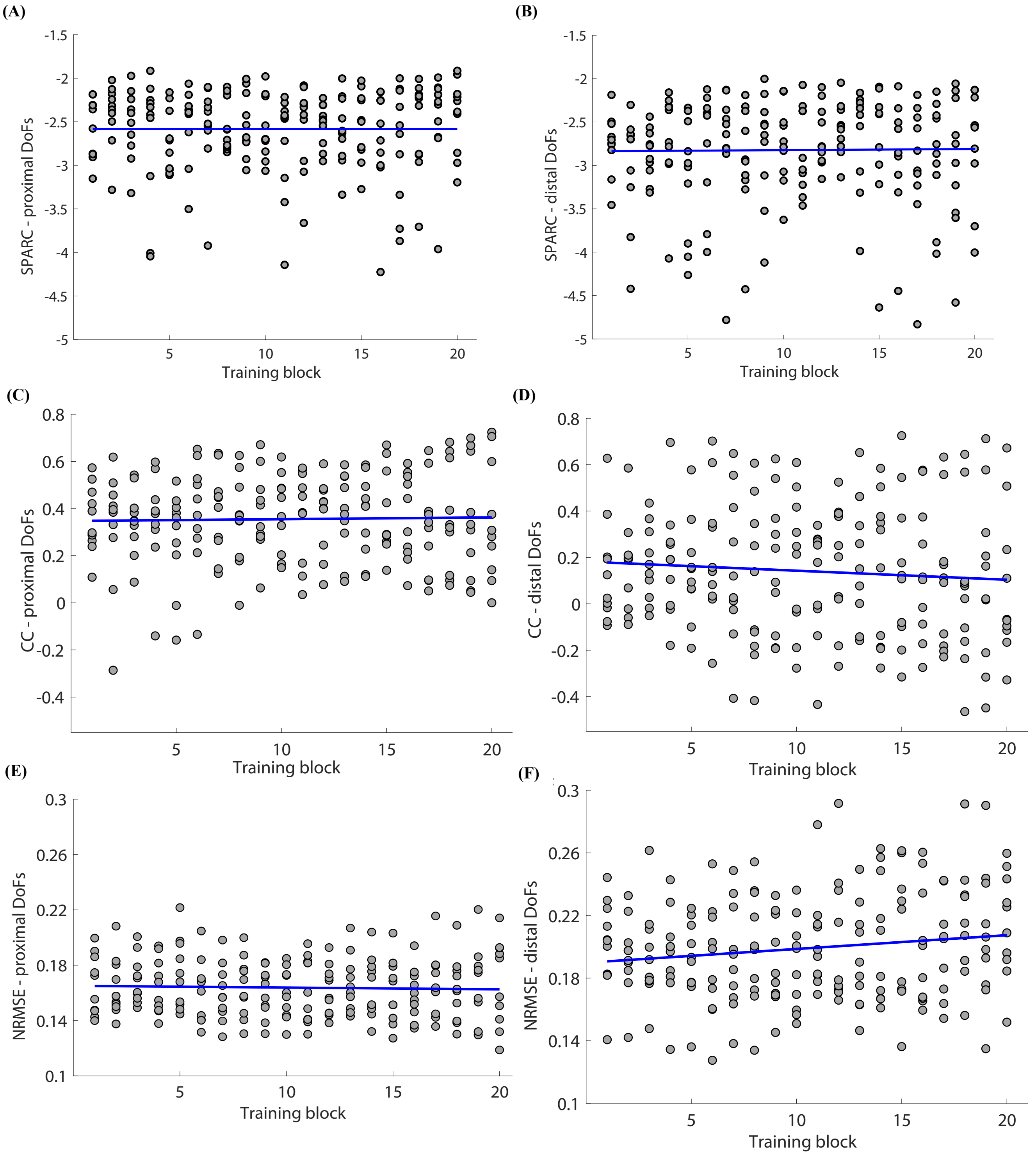

Supplementary Figure 2: SPARC (A)-(B), CC (C)-(D) and NRMSE (E)-(F) values obtained during the training blocks across sessions and linear model fitted to those values (blue line).
