# Supplementary material for: Real-Time Control of a Multi-Degree-of-Freedom Mirror Myoelectric Interface During Functional Task Training
Source: Front Neurosci. 2022 Mar 11;16:764936. doi: 10.3389/fnins.2022.764936 (PMC8962619; doi:10.3389/fnins.2022.764936)
Supplement: Supplementary file 3 [file Data_Sheet_3.pdf]

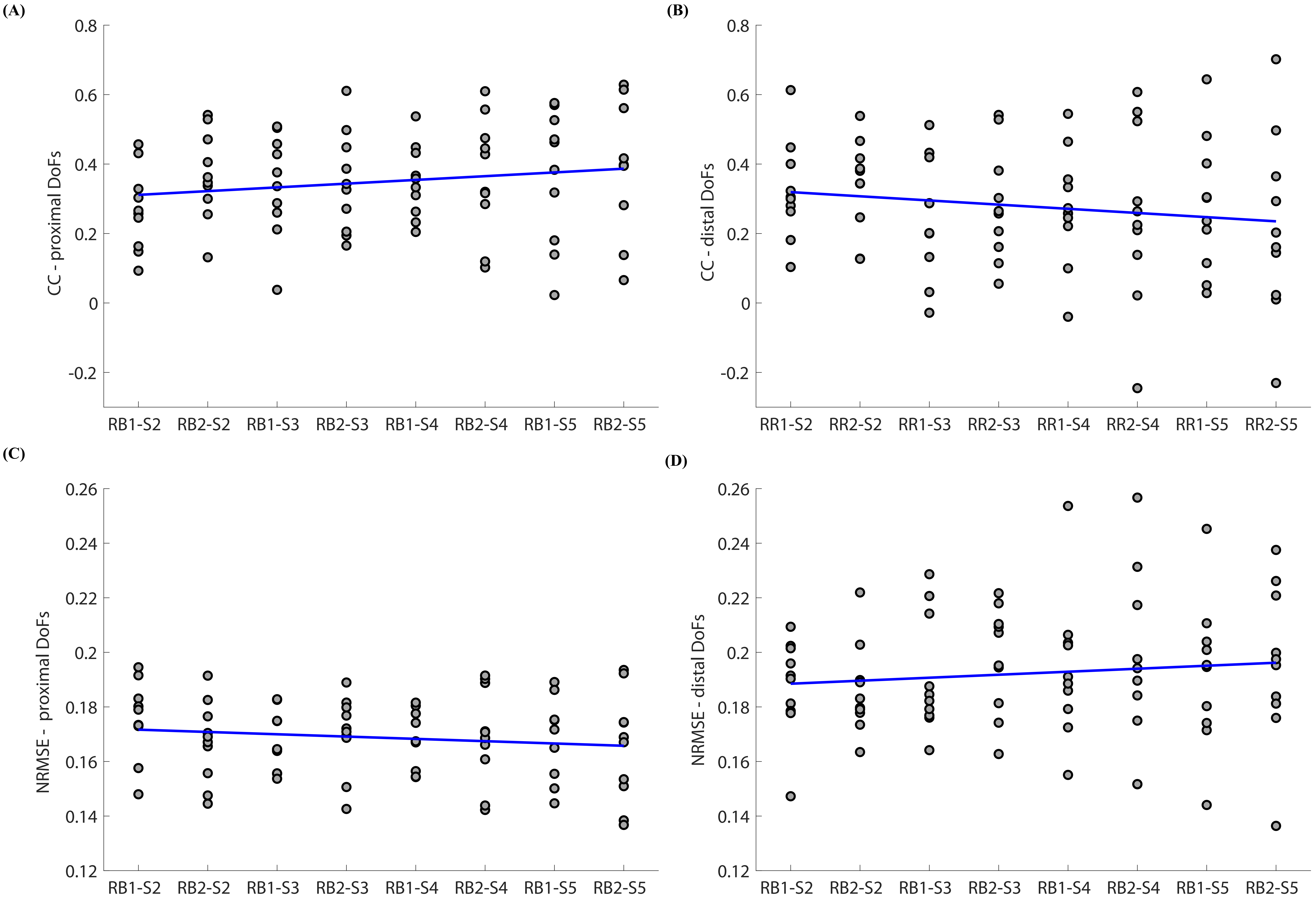

Supplementary Figure 3: CC (A)-(B) and NRMSE (C)-(D) values obtained during the reference blocks across sessions and linear model fitted to those values (blue line).
