# Supplementary material for: Real-Time Control of a Multi-Degree-of-Freedom Mirror Myoelectric Interface During Functional Task Training
Source: Front Neurosci. 2022 Mar 11;16:764936. doi: 10.3389/fnins.2022.764936 (PMC8962619; doi:10.3389/fnins.2022.764936)
Supplement: Supplementary file 4 [file Data_Sheet_4.pdf]

EMG-BASED CONTROL OF A 7-DOF EXOSKELETON

Feedback questionnaire

We would like you to rate several questions concerning the last training session.
On the following pages you´ll find some statements and questions related to your experience moving and controlling the exoskeleton. The questionnaire should evaluate how easy/difficult it was to control the movement of the exoskeleton with your muscle activity. Furthermore the self-rating of your movement performance and the sensations when you moved your arm together with the exoskeleton will be evaluated.
Please decide for every statement, how well it fits to you. There are no correct or wrong answers, please answer spontaneously. If you are uncertain about an answer, please choose the answer that´s fitting the best. We are only interested in your subjective impression.

Personal data:

Name: ____________________ **Last name:** ______________________________

Age: ______________________ **Sex:** ☐ m. ☐ f.

Session number: ___________

The numbers have following meaning:<br>⓪ = „applies absolutely not"<br>⑩ = „applies completely "		
1.	I understood the instructions completely, there were no uncertanties.	⓪ ① ② ③ ④ ⑤ ⑥ ⑦ ⑧ ⑨ ⑩
2.	I had the feeling that the exoskeleton assisted the movement I was trying to do.	⓪ ① ② ③ ④ ⑤ ⑥ ⑦ ⑧ ⑨ ⑩
3.	I had the feeling that the exoskeleton went against the movement I was trying to perform.	⓪ ① ② ③ ④ ⑤ ⑥ ⑦ ⑧ ⑨ ⑩
4.	The exoskeleton moved without interruptions.	⓪ ① ② ③ ④ ⑤ ⑥ ⑦ ⑧ ⑨ ⑩
5.	The exoskeleton moved at a comfortable speed.	⓪ ① ② ③ ④ ⑤ ⑥ ⑦ ⑧ ⑨ ⑩
6.	The exoskeleton moved always into the right direction.	⓪ ① ② ③ ④ ⑤ ⑥ ⑦ ⑧ ⑨ ⑩
7.	The movement of the exoskeleton supported me to find the correct strategy for the movement.	⓪ ① ② ③ ④ ⑤ ⑥ ⑦ ⑧ ⑨ ⑩
8.	I´ve been able to perform the hand/arm movement easily and smoothly	⓪ ① ② ③ ④ ⑤ ⑥ ⑦ ⑧ ⑨ ⑩
9.	I found it easy to control the base of the exoskeleton (i.e. movements of the upper- and fore- arm without the hand/wrist)	⓪ ① ② ③ ④ ⑤ ⑥ ⑦ ⑧ ⑨ ⑩
10.	I found it easy to control the hand module of the exoskeleton (i.e. open/close the fingers and pronate/supinate the wrist)	⓪ ① ② ③ ④ ⑤ ⑥ ⑦ ⑧ ⑨ ⑩
11.	I was physically tired after the experiment (i.e. my muscles were fatigued).	⓪ ① ② ③ ④ ⑤ ⑥ ⑦ ⑧ ⑨ ⑩
12.	I could keep the concentration to control the exoskeleton actively during the whole experiment (all runs).	⓪ ① ② ③ ④ ⑤ ⑥ ⑦ ⑧ ⑨ ⑩
13.	I felt mentally tired after the session.	⓪ ① ② ③ ④ ⑤ ⑥ ⑦ ⑧ ⑨ ⑩

14.	The breaks between runs were sufficient.	⓪ ① ② ③ ④ ⑤ ⑥ ⑦ ⑧ ⑨ ⑩
15.	The exoskeleton was comfortable (i.e. the parts in contact with my arm were smooth and soft).	⓪ ① ② ③ ④ ⑤ ⑥ ⑦ ⑧ ⑨ ⑩
16.	Wearing the exoskeleton was exhausting because I had to keep an uncomfortable body and arm posture during the experiment.	⓪ ① ② ③ ④ ⑤ ⑥ ⑦ ⑧ ⑨ ⑩
17.	I could perform the movements with the exoskeleton naturally.	⓪ ① ② ③ ④ ⑤ ⑥ ⑦ ⑧ ⑨ ⑩
18.	I had the feeling that the experimenters are well trained.	⓪ ① ② ③ ④ ⑤ ⑥ ⑦ ⑧ ⑨ ⑩
19.	The experimenters answered my questions clearly and acted professionally.	⓪ ① ② ③ ④ ⑤ ⑥ ⑦ ⑧ ⑨ ⑩
20.	The experiment ran smoothly and without technical interruptions.	⓪ ① ② ③ ④ ⑤ ⑥ ⑦ ⑧ ⑨ ⑩

Are there other noteworthy comments you´d like to make?
